# Association of Blood Amyloid Beta-Protein 1-42 with Poststroke Cognitive Impairment: A Systematic Review and Meta-Analysis

**DOI:** 10.1155/2022/6552781

**Published:** 2022-03-30

**Authors:** Hui Chen, Sichun Gu, Xiaoying Liu, Anjie Xie, Changde Wang

**Affiliations:** ^1^Department of Neurology, Shanghai Traditional Chinese Medicine Integrated Hospital, Shanghai University of Traditional Chinese Medicine, Shanghai, China; ^2^Department of Neurology, Longhua Hospital, Shanghai University of Traditional Chinese Medicine, 725 South Wanping Road, Shanghai, China

## Abstract

**Background:**

Increases in blood of amyloid beta-protein (A*β*) have been noted in patients with Alzheimer's dementia (AD). Recent studies have shown that blood amyloid beta-protein 1-42 (A*β*1-42) level is closely related to poststroke cognitive impairment (PSCI), which may be the influencing factor and even a predictor of PSCI. The aim of this systematic review was to synthesize the evidence for the association of cognitive impairment among PSCI.

**Methods:**

PubMed (MEDLINE), EMBASE, Cochrane Library, the Cochrane Central Register of Controlled Trial (CENTRAL), CNKI, and WanFang data were searched. Case-control, cohort, and cross-sectional studies that evaluated the association between blood A*β*1-42 and PSCI were included irrespective of language and date of publication. The outcomes of this review consisted of (1) any dementia, (2) any cognitive impairment, and (3) any cognitive impairment no dementia, which were assessed at least 3 months (90 days) after stroke. Exposure of interest was blood A*β*1-42 level (including serum and plasma).

**Results:**

Of 617 records retrieved, 8 studies (6 case-control and 2 cohort studies) involving 931 stroke patients were included for further analysis. 8 studies with 931 subjects explored the correlation between A*β*1-42 and PSCI. PSCI was reported in 457 patients, and the pooled SMD of amyloid beta-protein 1-42 was -0.96 (95% CI -1.10~-0.82, *I*^2^ = 15%, *P* < 0.01). The results remained robust in sensitivity analysis adjusting for study quality, sample source, and cognitive scale score in analysis of studies, as well as in analysis adjusted for publication bias.

**Conclusions:**

Blood A*β*1-42 level was significantly negatively related to the risk for PSCI, and more prospective studies with large sample size are needed to define a precise threshold value of blood A*β*1-42 level to predict PSCI in the future. This study is registered with PROSPERO, registration number: CRD42021246165.

## 1. Introduction

Stroke is among the leading causes of disability and death worldwide. Stroke patients are under a significantly higher risk of cognitive impairment and dementia than the general population [1, 2]. Poststroke cognitive impairment (PSCI), as one of the major complications after stroke, is a subtype of vascular cognitive impairment (VCI). The prevalence of dementia within five years after stroke ranges from 10% to 30% [3]. The risk of cognitive impairment after stroke increases at least five to eight times [4].

At present, the assessment of PSCI is usually based on neuropsychological evaluations, such as the Montreal Cognitive Assessment (MoCA), Mini-Mental State Examination (MMSE), and other related scales, which are limited in terms of accuracy and objectivity and are prone to the influence of age and education. It is difficult to perform accurate screening with a single application, and the combination of multiple scales will increase the burden on patients. Thus, neuropsychological tests seem not to be sufficient for the reliable prognosis and diagnosis of PSCI. In recent years, a number of studies have reported that biomarkers in circulating blood serum, plasma, and cerebrospinal fluid (CSF) of PSCI patients might be key determinants for the diagnosis and prediction of cognitive impairment.

Recent studies found that PSCI was closely related to the core pathological changes of Alzheimer's disease, suggesting that both of them may have identical diagnostic biomarkers [5, 6]. Amyloid beta-protein (A*β*) is the main component of senile plaques characterized by Alzheimer's disease [7]. Previous studies have suggested that serum A*β* might be an independent cerebrovascular risk factor, which can indicate vascular injury during dementia [8, 9]. Yu et al. [10] revealed that A*β* did not only exist in Alzheimer's disease but also was correlated with stroke progression. Furthermore, it was found that the deposition of plasma or serum amyloid beta-protein 1-42(A*β*1-42) was related to vascular risk factors, which suggested a good application prospect in prompting diagnosis and judgment of the severity of PSCI. Therefore, we performed a systematic review and meta-analysis to summarize relevant findings from observational studies and investigate the relationship between blood A*β*1-42 and cognitive impairment among patients after stroke.

## 2. Methods

### 2.1. Protocol Registration

The protocol of this study was prospectively registered at PROSPERO [https://www.crd.york.ac.uk/PROSPERO/(Registration number: CRD42021246165)] and reported in accordance with the PRISMA [11] and MOOSE [12] guidelines.

### 2.2. Literature Searches

Our latest search was conducted in the electronic literature databases: PubMed (MEDLINE), EMBASE, Cochrane Library, the Cochrane Central Register of Controlled Trial (CENTRAL), CNKI, and WanFang data from the inception to 31 March 2021. A combination of the three following groups of text or MeSH terms constituted the search strategies: (1) stroke and cerebrovascular disease; (2) amyloid beta-protein 1-42; and (3) cognitive impairment, dementia, and cognitive dysfunction. We searched completed studies registered on http://ClinicalTrial.gov in March 2021. We also scanned the reference lists of relevant systematic reviews and eligible primary studies. The detailed search strategy is described in supplemental Table 1.

### 2.3. Inclusion Criteria

We included eligible all observational studies conducted among patients with stroke and examining the association of A*β*1-42 with the outcomes of interest, regardless of study time, location, settings, data sources, sample size, stroke subtype, study quality, confounders adjusted, publication type, and language. Case reports, animal studies, autopsy studies, and studies on test of A*β*1-42 in cerebrospinal fluid were excluded. Our target population was adult (≥18 years) patients who have suffered stroke including ischemic stroke, hemorrhagic stroke, and TIA.

The outcomes of interest in this review consisted of (1) any dementia, (2) any cognitive impairment, and (3) any cognitive impairment no dementia. Exposures of interest were blood A*β*1-42level (including serum and plasma). The outcome should be assessed at least 3 months (90 days) after stroke and should be defined according to pre-specified criteria.

### 2.4. Study Selection

Two reviewers independently scanned the title and abstract based on the inclusion criteria. Then, we assessed the full-text articles for eligibility. A third reviewer approved the studies selected. When the data were missing, the principal investigators were contacted for further information. If the principal investigator could not provide the missing data, the study was excluded. A flowchart of study inclusion was developed in the format recommended by the PRISMA statement.

### 2.5. Data Extraction

Data from all eligible studies were extracted by two independent reviewers. The data extracted from each of the eligible studies included the first author, year of publication, study design, study region, population source, sample size, length of follow-up, mean age, number of cases, scale scores, risk factors adjusted, sample source (serum/plasma), and study quality. If risk estimates were not available, the authors were contacted for further information.

### 2.6. Quality Assessment

The assessment for the quality of observational studies in the meta-analysis was performed using the Newcastle-Ottawa Scale (NOS) [13]. The quality of studies was determined according to the selection criteria for participants, comparability of cases and controls, and exposure and outcome assessments. Quality score ranges from 0 to 9 points. On the whole, a score ≥5 indicated adequate quality for inclusion in the present review.

### 2.7. Statistical Analysis

All data were analyzed using the Review Manager (version 5.2, Copenhagen: the Nordic Cochrane Centre, the Cochrane Collaboration, 2012) and STATA 12.0 (StataCorp LP, Lakeway Drive College Station, TX, 77845, USA). The effect of A*β*1-42 level on the risk for poststroke cognitive impairment was evaluated on the basis of included studies. Standard mean deviation (SMD) and 95% confidence interval (CI) were used to evaluate the pooled effect. The *I*^2^ statistic and *χ*^2^ test were employed to assess the variability across studies attributable to heterogeneity beyond chance. *I*^2^ exceeding 50% was considered as moderate and high heterogeneity, while a *P* value greater than 0.10 in the *χ*^2^ test was interpreted as low-level heterogeneity [14]. A pooled effect was calculated with a fixed-effects model when there was no statistically significant heterogeneity; otherwise, a random effects model was employed [15]. A value of two-sided *P* value less than 0.05 was considered statistically significant. Subgroup was used to determine the robustness across different groups. Publication bias was evaluated by Egger test and funnel plot. Trim and fill method was used for adjustment once bias was present [16].

### 2.8. Sensitivity Analysis

Sensitivity analysis was conducted with only including studies using MoCA for the outcome of any cognitive impairment, which was a cut-off commonly used in epidemiological studies on cognitive impairment.

## 3. Results

### 3.1. Study Inclusion Characteristics

**Figure 1:**
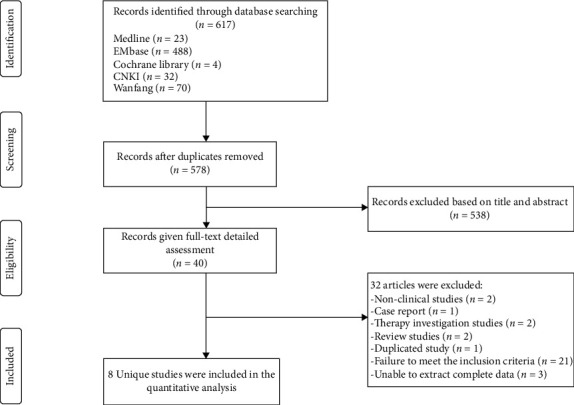
Flowchart of the study collection for the present review and meta-analysis.

A total of 617 studies were identified after initial searching. After reviewing the title and abstract, the full texts of 40 studies were obtained, in which 8 studies were included for further analysis and 32 studies were excluded because they did not meet the inclusion criteria (Figure 1). In 8 studies, there were 2 cohort studies and 6 case-control studies. Of these studies, there were 931 subjects; sample size varied from 55 to 188. The mean age of patients ranged from 61 to 68 years, and the length of follow-up ranged from 8 to 33 month. Details about exposure and outcomes in each included study are shown in Table 1.

### 3.2. Correlation between Blood Amyloid Beta-Protein 1-42 Level and Poststroke Cognitive Impairment

**Figure 2:**
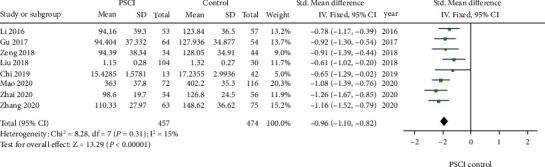
Studies on the association of blood A*β*1-42 level with PSCI.

In 8 studies, the correlation between blood A*β*1-42 level and PSCI was investigated. There were 6 case-control and 2 cohort studies with involvement of 931 subjects. In these studies, PSCI was reported in 457 patients. There was low heterogeneity among 8 studies (*I*^2^ = 15%, *P* = 0.31), and thus, fixed-effects model was used for further analysis. The pooled SMD of A*β*1-42 was -0.96 (95% CI -1.10~ -0.82, *I*^2^ = 15%, *P* < 0.01, Figure 2), suggesting that blood A*β*1-42 level in patients after stroke with cognitive impairment was significantly lower than those in patients without PSCI. The references for each study have been listed in Supplement Table 2.

### 3.3. Publication Bias

**Figure 3:**
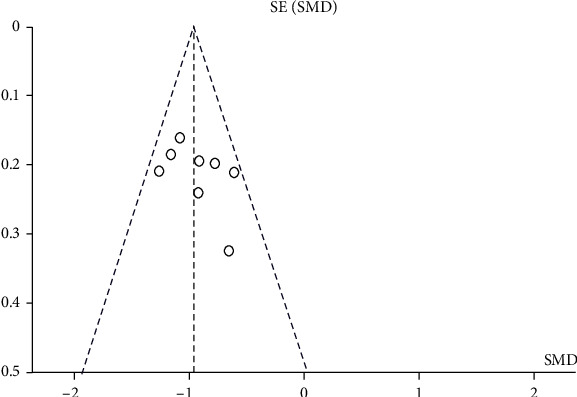
Funnel plot of the association of blood amyloid beta-protein (1-42) level in PSCI group and control group.

**Figure 4:**
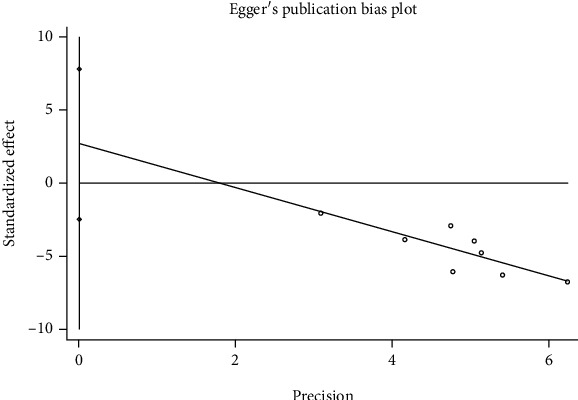
Egger's test for the association of blood amyloid beta-protein (1-42) level in PSCI group and control group.

The funnel plot was used to test the publication bias, and the graph is roughly symmetrical, which prompted that there was no obvious publication bias (Figure 3). Similarly, results of the Egger's test for PSCI (*P* = 0.251) suggested that any publication bias across included studies was unlikely (Figure 4).

### 3.4. Sensitivity Analysis

**Figure 5:**
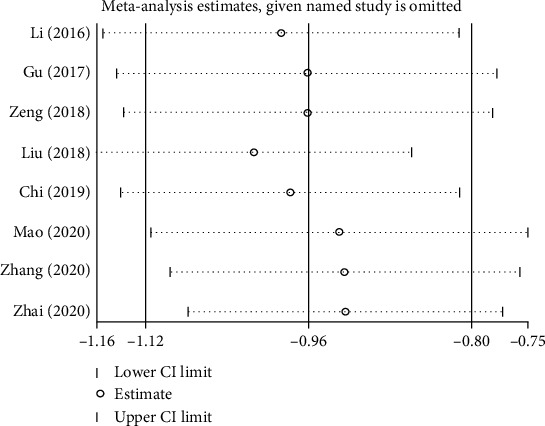
Sensitivity analysis for the association of blood amyloid beta-protein 1-42 level in PSCI group and control group.

Using STATA for sensitivity analysis, the result showed that there was small heterogeneity between the studies, so the results of our study are considered stable and reliable (Figure 5). Lower CI Limit represents the lower 95% CI limit for SMD, Upper CI Limit represents the upper 95% CI limit for SMD, and Estimate represents the point estimate, representing the pooled standardized mean difference for the remaining studies after this study was removed.

### 3.5. Subgroup Analysis

A subgroup analysis was conducted based on study quality (NOS), sample source (serum or plasma), and scale score (MoCA) for all outcomes. The results demonstrated that no subgroup effects in all outcomes. The details are shown in Supplemental Figures.

## 4. Discussion

These systematic review and meta-analysis were conducted to systemically investigate the relationship between blood A*β*1-42 level and poststroke cognitive impairment of available observational studies.

There is increasing evidence that the occurrence of PSCI is very common and is related to the onset of dementia [17]. PSCI not only severely affects the patient's ability of daily living [18], but also has a certain impact on the recovery of patients' physical function [19]. The highest risk of PSCI occurs 3 to 6 months after stroke, and cognitive impairment may persist [20]. Currently, the most commonly used assessment for detecting cognitive impairment include psychological test scale, imaging examinations, cerebrospinal fluid and blood biomarkers, and other related examinations. The neuropsychological test scale examination in the acute phase of stroke will be affected by many factors, such as consciousness, psychological factors and headaches, and motor and visual and speech dysfunctions [21]. Moreover, the assessment of the scale is subjective and requires a high degree of professional knowledge and training, which limits its routine clinical application. Neuropsychological imaging examinations related to cognitive impairment (such as PET-CT imaging examinations) are expensive, and are limited by the level of facilities in medical institutions, and cannot be generally used for pre-clinical screening.

Thus, it is of great practical significance to find simple and noninvasive biomarkers to diagnose and predict PSCI. Up to now, the amyloid cascade hypothesis has been widely considered to underlie the pathogenesis in AD [22]. A*β* in the brain is formed by continuous hydrolysis of its precursor substance beta amyloid precursor protein (amyloid precursor protein, APP), which mainly contains two forms of A*β*1-40 and A*β*1-42 [23]. Previous studies demonstrated a close relation between plasma A*β*1-40 and A*β*1-42 levels with AD [24, 25]. Furthermore, the National Institute of Aging and the Alzheimer's Society included A*β*1-42 in the new diagnostic criteria for AD dementia in 2011, which helped shape a new biomarker for PSCI. Consequently, in the current study, six case-control studies and two cohort studies with 931 subjects were included to explore the potential role of the blood A*β*1-42 for PSCI. And we found that the blood A*β*1-42 level in patients with PSCI was significantly lower than those without PSCI, showing that the blood A*β*1-42 level was negatively correlated with PSCI. Thus, our study has identified a significant link between plasma A*β*1-42 level with PSCI, which suggested that A*β*1-42 level might be a valuable biomarker for PSCI.

The underlying mechanism of A*β*1-42 level for PSCI has been explored in some literatures. Based on experimental studies, it is considered that acute ischemia increases A*β* precursor protein expression and A*β* production shortly after ischemic injury, thereby enhancing A*β* deposition and clearance in brain [26]. Goulay et al. [27] speculated that impaired perivascular space integrity, inflammation, hypoxia, and blood–brain barrier (BBB) breakdown after stroke can lead to accelerated deposition of A*β* within brain parenchyma and cerebral vessel walls or exacerbation of cerebral amyloid angiopathy (CAA). The deposition of A*β* in the parenchyma would then be the initiating event leading to synaptic dysfunction, inducing cognitive decline and dementia. Moulin et al. [28] supposed that plasma A*β*1-40 being involved in vascular aspects whereas A*β*1-42 might be involved in neurodegenerative processes. There is an A*β*1-42 deposition in the brain tissue of stroke animal models and patients with vascular dementia, which might be related to the induction of nitric oxide, protein, and membrane damage [29].

Consistent with our findings that A*β*1-42 level is meaningful for predicting PSCI, Chi et al. [30] found that low plasma A*β*1-42 level at 3 months was the most significant predictor of PSCI at 1 year. Mao et al. [31] also demonstrated lower serum A*β*1-42 level in patients with PSCI. When a patient suffered from stroke, the A*β*1-42 in the serum is significantly reduced due to the precipitation effect [32–34]. Cerebral ischemia-reperfusion injury can induce neuronal damage in the hippocampus and temporal cortex in an A*β*-dependent manner [35]. Low serum A*β*1-42 concentration is an independent risk factor for ischemic PSCI, and the serum A*β*1-42 level is positively correlated with the level of cognitive function after stroke [36]. Contrary to our results, Tang et al. [37] reported that plasma level of A*β*1-42 remained significantly lower in stroke patients with dementia than those without dementia after adjustment for age, sex, diabetes mellitus, hypertension, and hyperlipidemia. The difference in the time point for evaluation might explain the conflicting results to some extent, as circulating A*β* levels would dynamically change. Some misinterpretation of A*β* concentrations can also be due to varying ELISA protocols and different A*β* antibodies with inconsistent sensitivity and specificity [38].

There were several strengths in our study. First, most of the included studies were adjusted potential risk factors, and the methodological evaluation was satisfactory. Secondly, various studies on the relationship between A*β*1-42 and poststroke cognitive impairment had low heterogeneity and no publication bias, indicating that the results of the meta-analysis were reliable. Limitations should be noted. The sample size of each included study was small (maximum 188 cases, minimum 55 cases). The follow-up time was relatively short (up to 2.75 years). Only Asian populations were included in the studies, lacking of data for populations such as Europe and America. In addition, there were few studies on the relationship between A*β*1-42 level and cognitive impairment after hemorrhagic stroke, which limits the elucidation of relationship of A*β* with stroke of different types. Lastly, the Web of Science and Scopus articles were not included in our study.

## 5. Conclusions

In summary, the case-control studies and cohort studies included in this analysis confirmed the negative relationship between blood A*β*1-42 level and PSCI, as well as supporting the expectations that blood A*β*1-42 level might be a meaningful biomarker for PSCI. From the standpoint of clinicians, it would be prudent to define a precise threshold value of blood A*β*1-42 level to predict PSCI. Thus, more prospective studies with large sample size are needed to allow translation into clinical practice in the future.

## Figures and Tables

**Table 1:** Characteristics of included studies.

Study ID	Design	Region	Setting	Sample size	No. of cases	Male (%)	Starting and ending time	Length of follow-up (years)	Scale score (diagnostic criteria)	Age (years) (range or mean ± SD)	Risk factors adjusted	Blood sample	Study quality (NOS)
Li et al. (2016) [36]	Case control	Xinjiang, China	Hospital/community	110	53	0.63	2014.12-2015.8	0.75	MoCA<26	66.49 ± 11.63	Age, education level, hypertension, diabetes, smoke, alcohol, TC, TG, LDL, HDL, UA, Hcy, CRP	Serum	8
Gu et al. (2017)	Case control	Xinjiang, China	Hospital/community	118	64	NA	2016.11-2017.6	0.67	MoCA<26		NA	Serum	6
Zeng et al. (2018)	Case control	Hubei, China	Hospital	78	34	0.54	2016.1-2018.1	2.00	MoCA<26		NA	Serum	5
Liu et al. (2018)	Case control	Hubei, China	Hospital/community	134	104	0.51	2013.7-2016.3	2.75	MoCA<26	67.5 ± 10.8	Age, gender	Serum	7
Chi et al. (2019) [30]	Cohort	Taipei, China	Hospital	55	13	0.82		1	MoCA<23	61.18 ± 7.93	Age, education level, hypertension, diabetes mellitus, NIHSS, Fazekas scale	Plasma	8
Mao et al. (2020) [31]	Cohort	Shanghai, China	Hospital	188	72	0.62	2016.6-2018.1	1.67	MoCA<26	68.06 ± 11.05	Age, gender, education level	Serum	7
Zhang et al. (2020)	Case control	Henan, China	Hospital/community	138	63	0.62	2019.1-2019.12	1	MoCA<26	63.29 ± 8.12	Age, gender, education level, hypertension, diabetes, smoke, NIHSS	Serum	8
Zhai et al. (2020)	Case control	Henan, China	Hospital	110	54	0.62	2017.1-2018.2	1.17	MoCA<26	64.90 ± 8.68	Age, gender, BMI, education level, hypertension, diabetes, CAD, smoke	Serum	7

TC: total cholesterol; TG: triglycerides; LDL: low-density lipoprotein; HDL: high-density lipoprotein; UA: uric acid; Hcy: homocysteine; CRP: C-reactive protein; N/A: nonavailable; NIHSS: National Institutes of Health Stroke Scale; CAD: coronary atherosclerotic disease.

## Data Availability

All the datasets used in this study are public databases.

## Supplementary Materials

Supplementary Materials Supplementary Figure 1. Subgroup analysis based on high quality (>6) versus medium quality (≤6) of NOS. Supplementary Figure 2. Subgroup analysis based on serum versus plasma. Supplementary Figure 3. Subgroup analysis based on higher score (<26) versus lower score (<23) of MoCA. Supplementary Table 1. Search strategy
