# Supplementary material for: Association of Blood Amyloid Beta-Protein 1-42 with Poststroke Cognitive Impairment: A Systematic Review and Meta-Analysis
Source: Biomed Res Int. 2022 Mar 30;2022:6552781. doi: 10.1155/2022/6552781 (PMC8986382; doi:10.1155/2022/6552781)
Supplement: Supplementary Materials — Supplementary Figure 1. Subgroup analysis based on high quality (>6) versus medium quality (≤6) of NOS. Supplementary Figure 2. Subgroup analysis based on serum versus plasma. Supplementary Figure 3. Subgroup analysis based on higher score (<26) versus lower score (<23) of MoCA. Supplementary Table 1. Search strategy [file 6552781.f1.pdf]

Supplementary Material

Association of blood amyloid beta-protein 1-42 with post-stroke cognitive impairment: a systematic review and meta-analysis

Supplementary Figures

Supplementary Figure 1. Subgroup (NOS)

Study or Subgroup	PSCI Mean	PSCI SD	PSCI Total	Control Mean	Control SD	Control Total	Weight	Std. Mean Difference IV, Fixed, 95% CI	Year
**1.3.1 NOS＞6**									
Li 2016	94.16	39.3	53	123.84	36.5	57	13.2%	-0.78 [-1.17, -0.39]	2016
Liu 2018	1.15	0.28	104	1.32	0.27	30	11.7%	-0.61 [-1.02, -0.20]	2018
Chi 2019	15.4285	1.5781	13	17.2355	2.9936	42	5.0%	-0.65 [-1.29, -0.02]	2019
Mao 2020	363	37.8	72	402.2	35.3	116	20.3%	-1.08 [-1.39, -0.76]	2020
Zhai 2020	98.6	19.7	54	126.8	24.5	56	11.9%	-1.26 [-1.67, -0.85]	2020
Zhang 2020	110.33	27.97	63	148.62	36.62	75	15.2%	-1.16 [-1.52, -0.79]	2020
**Subtotal (95% CI)**			**359**			**376**	**77.3%**	**-0.97 [-1.13, -0.81]**	
Heterogeneity: Chi² = 8.18, df = 5 (P = 0.15); I² = 39%									
Test for overall effect: Z = 11.83 (P < 0.00001)									
**1.3.2 NOS≤6**									
Gu 2017	94.404	37.332	64	127.936	34.877	54	13.7%	-0.92 [-1.30, -0.54]	2017
Zeng 2018	94.39	38.34	34	128.05	34.91	44	9.0%	-0.91 [-1.39, -0.44]	2018
**Subtotal (95% CI)**			**98**			**98**	**22.7%**	**-0.92 [-1.21, -0.62]**	
Heterogeneity: Chi² = 0.00, df = 1 (P = 0.99); I² = 0%									
Test for overall effect: Z = 6.07 (P < 0.00001)									
**Total (95% CI)**			**457**			**474**	**100.0%**	**-0.96 [-1.10, -0.82]**	
Heterogeneity: Chi² = 8.28, df = 7 (P = 0.31); I² = 15%									
Test for overall effect: Z = 13.29 (P < 0.00001)									
Test for subgroup differences: Chi² = 0.09, df = 1 (P = 0.76), I² = 0%									

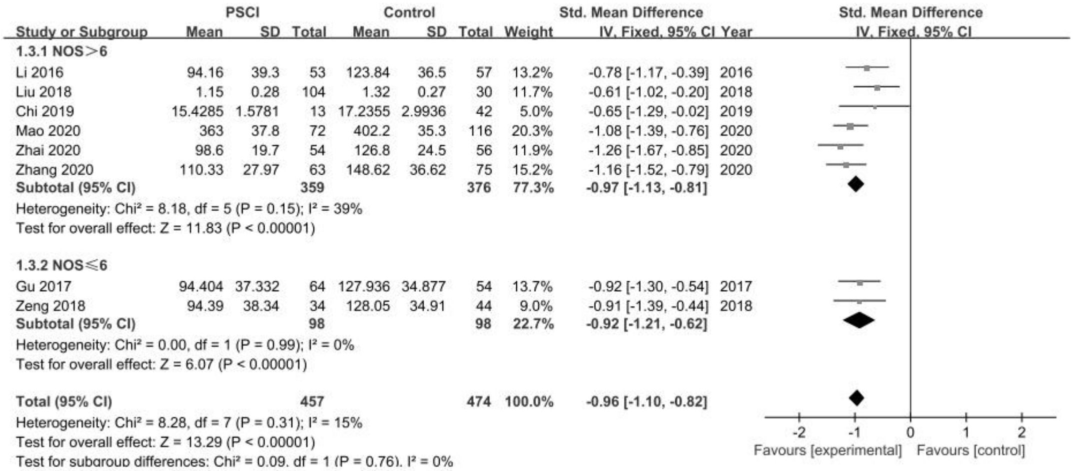

Supplementary Figure 2. Subgroup (serum, plasma)

Study or Subgroup	PSCI Mean	PSCI SD	PSCI Total	Control Mean	Control SD	Control Total	Weight	Std. Mean Difference IV, Fixed, 95% CI	Year
**1.1.1 serum**									
Li 2016	94.16	39.3	53	123.84	36.5	57	13.2%	-0.78 [-1.17, -0.39]	2016
Gu 2017	94.404	37.332	64	127.936	34.877	54	13.7%	-0.92 [-1.30, -0.54]	2017
Zeng 2018	94.39	38.34	34	128.05	34.91	44	9.0%	-0.91 [-1.39, -0.44]	2018
Liu 2018	1.15	0.28	104	1.32	0.27	30	11.7%	-0.61 [-1.02, -0.20]	2018
Zhai 2020	98.6	19.7	54	126.8	24.5	56	11.9%	-1.26 [-1.67, -0.85]	2020
Mao 2020	363	37.8	72	402.2	35.3	116	20.3%	-1.08 [-1.39, -0.76]	2020
Zhang 2020	110.33	27.97	63	148.62	36.62	75	15.2%	-1.16 [-1.52, -0.79]	2020
**Subtotal (95% CI)**			**444**			**432**	**95.0%**	**-0.97 [-1.12, -0.83]**	
Heterogeneity: Chi² = 7.33, df = 6 (P = 0.29); I² = 18%									
Test for overall effect: Z = 13.17 (P < 0.00001)									
**1.1.2 plasma**									
Chi 2019	15.4285	1.5781	13	17.2355	2.9936	42	5.0%	-0.65 [-1.29, -0.02]	2019
**Subtotal (95% CI)**			**13**			**42**	**5.0%**	**-0.65 [-1.29, -0.02]**	
Heterogeneity: Not applicable									
Test for overall effect: Z = 2.01 (P = 0.04)									
**Total (95% CI)**			**457**			**474**	**100.0%**	**-0.96 [-1.10, -0.82]**	
Heterogeneity: Chi² = 8.28, df = 7 (P = 0.31); I² = 15%									
Test for overall effect: Z = 13.29 (P < 0.00001)									
Test for subgroup differences: Chi² = 0.95, df = 1 (P = 0.33), I² = 0%									

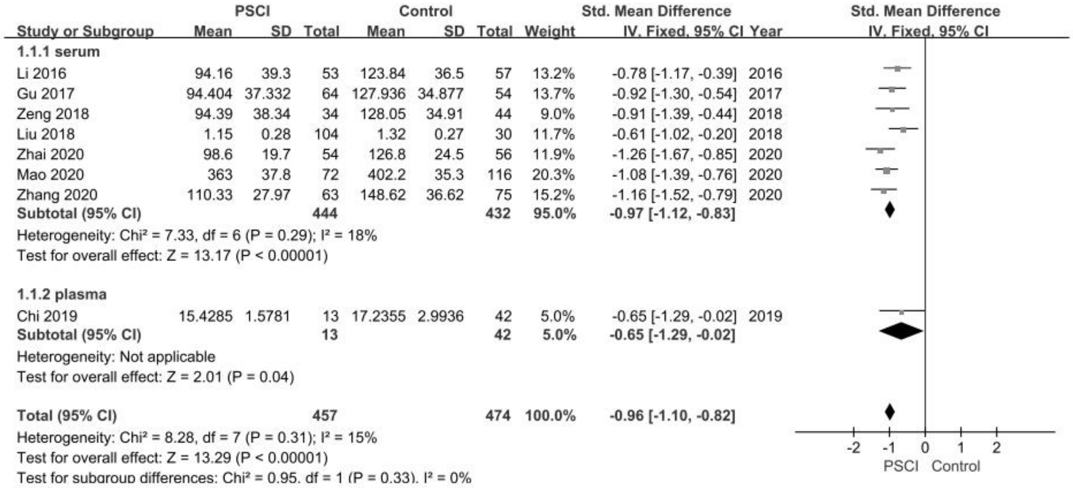

Supplementary Figure 3. Subgroup (MoCA)

Study or Subgroup	PSCI Mean	PSCI SD	PSCI Total	Control Mean	Control SD	Control Total	Weight	Std. Mean Difference IV, Fixed, 95% CI	Year
**1.1.2 MoCA＜26**									
Li 2016	94.16	39.3	53	123.84	36.5	57	13.2%	-0.78 [-1.17, -0.39]	2016
Gu 2017	94.404	37.332	64	127.936	34.877	54	13.7%	-0.92 [-1.30, -0.54]	2017
Liu 2018	94.39	38.34	34	128.05	34.91	44	9.0%	-0.91 [-1.39, -0.44]	2018
Zeng 2018	1.15	0.28	104	1.32	0.27	30	11.7%	-0.61 [-1.02, -0.20]	2018
Mao 2020	363	37.8	72	402.2	35.3	116	20.3%	-1.08 [-1.39, -0.76]	2020
Zhang 2020	110.33	27.97	63	148.62	36.62	75	15.2%	-1.16 [-1.52, -0.79]	2020
Zhai 2020	98.6	19.7	54	126.8	24.5	56	11.9%	-1.26 [-1.67, -0.85]	2020
**Subtotal (95% CI)**			**444**			**432**	**95.0%**	**-0.97 [-1.12, -0.83]**	
Heterogeneity: Chi² = 7.33, df = 6 (P = 0.29); I² = 18%									
Test for overall effect: Z = 13.17 (P < 0.00001)									
**1.1.3 MoCA＜23**									
Chi 2019	15.4285	1.5781	13	17.2355	2.9936	42	5.0%	-0.65 [-1.29, -0.02]	2019
**Subtotal (95% CI)**			**13**			**42**	**5.0%**	**-0.65 [-1.29, -0.02]**	
Heterogeneity: Not applicable									
Test for overall effect: Z = 2.01 (P = 0.04)									
**Total (95% CI)**			**457**			**474**	**100.0%**	**-0.96 [-1.10, -0.82]**	
Heterogeneity: Chi² = 8.28, df = 7 (P = 0.31); I² = 15%									
Test for overall effect: Z = 13.29 (P < 0.00001)									
Test for subgroup differences: Chi² = 0.95, df = 1 (P = 0.33), I² = 0%									

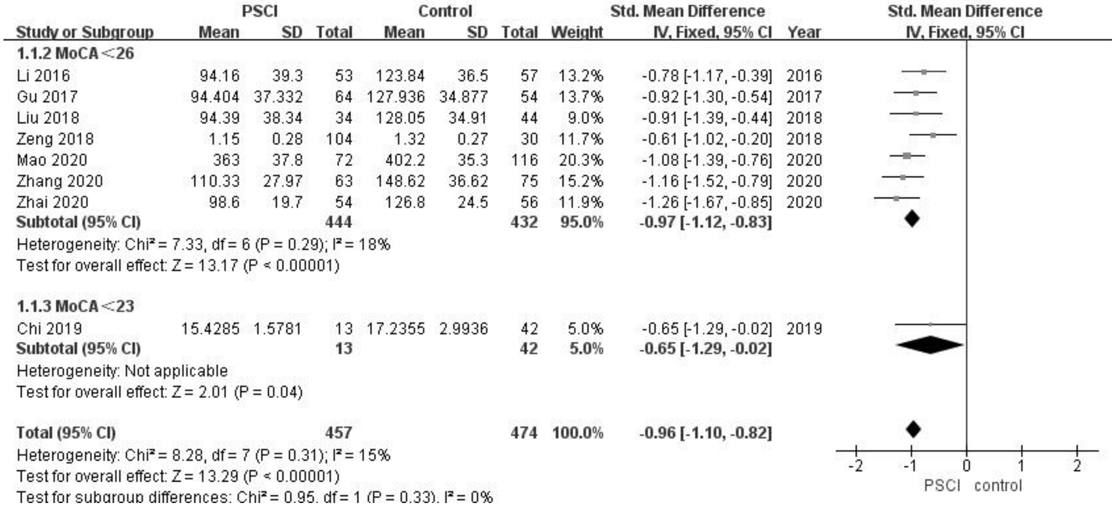

Supplementary Tables

Supplementary Table 1. Search Strategy

	**MEDLINE(Ovid), until 31 Mar. 2021**
1	"amyloid beta-protein (1-42)" [Supplementary Concept]
2	amyloid-beta-42 peptide[Title/Abstract] OR Abeta(1-42)[Title/Abstract] OR
	beta-amyloid (1-42)[Title/Abstract] OR Abeta42 protein[Title/Abstract]
3	1 or 2
4	"Stroke"[Mesh]
5	Strokes[Title/Abstract] OR Cerebrovascular Acciden*[Title/Abstract] OR
	CVA* (Cerebrovascular Accident)[Title/Abstract] OR Cerebrovascular
	Apoplexy[Title/Abstract] OR Apoplexy, Cerebrovascular[Title/Abstract] OR
	Vascular Acciden*, Brain[Title/Abstract] OR Brain Vascular
	Acciden*[Title/Abstract] OR Cerebrovascular Strok*[Title/Abstract] OR
	Strok*, Cerebrovascular[Title/Abstract] OR Apoplexy[Title/Abstract] OR
	Cerebral Strok*[Title/Abstract] OR Strok*, Cerebral[Title/Abstract] OR
	Strok*, Acute[Title/Abstract] OR Acute Strok*[Title/Abstract] OR
	Cerebrovascular Acciden*, Acute[Title/Abstract] OR Acute Cerebrovascular
6	Acciden*[Title/Abstract]
7	"Cerebral Infarction"[Mesh]
	Cerebral Infarctions[Title/Abstract] OR Infarctio*, Cerebral[Title/Abstract]
	OR Cerebral Infarc*[Title/Abstract] OR Infarc*, Cerebral[Title/Abstract]
	OR Subcortical Infarctio*[Title/Abstract] OR Infarctio*,
	Subcortical[Title/Abstract] OR Posterior Choroidal Artery
8	Infarction[Title/Abstract] OR Anterior Choroidal Artery
9	Infarction[Title/Abstract]
	"Cerebral Hemorrhage"[Mesh]
	Hemorrhag*, Cerebrum[Title/Abstract] OR Cerebrum
	Hemorrhag*[Title/Abstract] OR Cerebral Parenchymal
	Hemorrhag*[Title/Abstract] OR Hemorrhag*, Cerebral
	Parenchymal[Title/Abstract] OR Parenchymal Hemorrhag*,
	Cerebral[Title/Abstract] OR Intracerebral Hemorrhag*[Title/Abstract] OR
	Hemorrhag*, Intracerebral[Title/Abstract] OR Hemorrhag*,
10	Cerebral[Title/Abstract] OR Cerebral Hemorrhages[Title/Abstract] OR
11	Brain Hemorrhag*, Cerebral[Title/Abstract] OR Cerebral Brain
12	Hemorrhag*[Title/Abstract] OR Hemorrhag*, Cerebral
	Brain[Title/Abstract]
	4 OR 5 OR 6 OR 7 OR 8 OR 9
	"Cognitive Dysfunction"[Mesh]
	Cognitive Dysfunctions[Title/Abstract] OR Dysfunctio*,
	Cognitive[Title/Abstract] OR Cognitive Impairmen*[Title/Abstract] OR
13	Impairmen*, Cognitive[Title/Abstract] OR Mild Cognitive
14	Impairmen*[Title/Abstract] OR Cognitive Impairmen*,
	Mild[Title/Abstract] OR Impairmen*, Mild Cognitive[Title/Abstract] OR

	Cognitive Declin*[Title/Abstract] OR Declin*, Cognitive[Title/Abstract]<br>11 OR 12<br>3 AND 10 AND 13
 1<br>2<br>3<br>4<br>5<br>6<br>7<br>8<br>9<br>10<br>11<br>12<br>13	**EMBASE(Ovid), until 31 Mar. 2021**<br>'post stroke cognitive impairment'/exp<br>'post stroke dementia'/exp<br>'cerebrovascular accident'/exp<br>'brain infarction'/exp<br>'brain hemorrhage'/exp<br>3 OR 4 OR 5<br>'cognitive defect'/exp<br>'dementia'/exp<br>7 OR 8<br>6 AND 9<br>1 OR 2 OR 10<br>'amyloid beta protein 1 42'/exp<br>11 AND 12
 1	**CNKI, until 31 Mar. 2021**<br>SU=(卒中+脑梗死+脑出血)*(认知减退+认知障碍+痴呆)*(淀粉样蛋白 1-42)

List of included studies' references

1. Li M, Wang H, Wang MS. The correlation of serum Aβ1-42 concentration and cognitive impairment after ischemic stroke. *Modern Medicine Journal of China.* 2016;18(01):9-12. DOI:10.3969/j.issn.1672-9463.2016.01.003
2. Gu Y, Wang H, Li JM, *et al.* Serum soluble CD40 ligand, beta amyloid 1-42 level diagnostic efficacy of cognitive dysfunction after stroke. *Shandong Medical Journal*. 2017;57(43):49-51. DOI:10.3969/j.issn.1002-266X.2017.43.015
3. Zeng HM. Analysis of the value of serum soluble CD40 ligand (sCD40L) and β-amyloid 1-42 (Aβ1-42) results in the diagnosis of post-stroke cognitive impairment (PSCI). *World Latest Medicine Information*. 2018;18(58):193-194. DOI:10.19613/j.cnki.1671-3141.2018.58.104
4. Liu YY, Liu JJ, Wang DM. Relationship between serum Aβ1-42, hcy and tau protein levels in patients with vascular cognitive impairment after stroke. *Sichuan Medical Journal.* 2018;39(6): DOI:10.16252/j.cnki.issn1004-0501-2018.06.023
5. Chi NF, Chao SP, Huang LK, *et al.* Plasma amyloid beta and tau levels are predictors of post-stroke cognitive impairment: a longitudinal study. *Front Neurol.* 2019;10:715. DOI:10.3389/fneur.2019.00715
6. Mao L, Chen XH, Zhuang JH, *et al.* Relationship between beta-amyloid protein 1-42, thyroid hormone levels and the risk of cognitive impairment after ischemic stroke. *World J Clin Cases.* 2020;8(1):76-87. DOI:10.12998/wjcc.v8.i1.76
7. Zhang HL, Zhang YL, Chen GJ, *et al.* The correlation of serum Aβ1-42, uric acid concentration and transient ischemic attack and minor stroke patients' cognitive impairment. *Chinese Journal of Practical Nervous Disease.* 2020;23(12):1067-1071. DOI:10.12083/sysj.2020.12.244
8. Zhai XY. Relationship between changes of serum VEGF, VILIP1, sCD40L and Aβ1-42 proteins and cognitive impairment in patients with ACI. J Huaihai Med. 2020;38(5):453-456,460. DOI: 10.14126 /j.cnki.1008-7044.2020.05.004
